# Evaluating automated longitudinal tumor measurements for glioblastoma response assessment

**DOI:** 10.3389/fradi.2023.1211859

**Published:** 2023-09-07

**Authors:** Yannick Suter, Michelle Notter, Raphael Meier, Tina Loosli, Philippe Schucht, Roland Wiest, Mauricio Reyes, Urspeter Knecht

**Affiliations:** ^1^ ARTORG Center for Biomedical Engineering Research, University of Bern, Bern, Switzerland; ^2^ Department of Radiation Oncology, Inselspital, Bern University Hospital and University of Bern, Bern, Switzerland; ^3^ Cantonal Hospital of Graubünden, Chur, Switzerland; ^4^ Support Center for Advanced Neuroimaging, Inselspital, Bern, Switzerland; ^5^ Department of Neurosurgery, Inselspital, Bern, Switzerland; ^6^ Radiology Department, Spital Emmental, Burgdorf, Switzerland

**Keywords:** glioblastoma, segmentation, automated response assessment, RANO, tumor burden measurements, longitudinal

## Abstract

Automated tumor segmentation tools for glioblastoma show promising performance. To apply these tools for automated response assessment, longitudinal segmentation, and tumor measurement, consistency is critical. This study aimed to determine whether BraTumIA and HD-GLIO are suited for this task. We evaluated two segmentation tools with respect to automated response assessment on the single-center retrospective LUMIERE dataset with 80 patients and a total of 502 post-operative time points. Volumetry and automated bi-dimensional measurements were compared with expert measurements following the Response Assessment in Neuro-Oncology (RANO) guidelines. The longitudinal trend agreement between the expert and methods was evaluated, and the RANO progression thresholds were tested against the expert-derived time-to-progression (TTP). The TTP and overall survival (OS) correlation was used to check the progression thresholds. We evaluated the automated detection and influence of non-measurable lesions. The tumor volume trend agreement calculated between segmentation volumes and the expert bi-dimensional measurements was high (HD-GLIO: 81.1%, BraTumIA: 79.7%). BraTumIA achieved the closest match to the expert TTP using the recommended RANO progression threshold. HD-GLIO-derived tumor volumes reached the highest correlation between TTP and OS (0.55). Both tools failed at an accurate lesion count across time. Manual false-positive removal and restricting to a maximum number of measurable lesions had no beneficial effect. Expert supervision and manual corrections are still necessary when applying the tested automated segmentation tools for automated response assessment. The longitudinal consistency of current segmentation tools needs further improvement. Validation of volumetric and bi-dimensional progression thresholds with multi-center studies is required to move toward volumetry-based response assessment.

## 1. Introduction

Glioblastoma (GBM) is an intrinsic brain tumor ranking highest at grade IV on the World Health Organization’s malignancy scale. GBM patients have a median survival of only 16 months (1), making close monitoring of the disease path crucial. A patient’s response to therapy is evaluated based on the Response Assessment in Neuro-Oncology (RANO) criteria. RANO relies on a qualitative and quantitative evaluation of follow-up studies against a reference time point. The quantitative analysis focuses on the size of the contrast-enhancing tumor on post-contrast T1-weighted magnetic resonance imaging (MRI) (2–4). The qualitative assessment considers the T2/fluid-attenuated inversion recovery (FLAIR) abnormality on MRI. This text refers to the standard radiological RANO guidelines and does not consider variants such as immunotherapy RANO (iRANO) (5,6).

The gold standard of quantifying the tumor volume by manual segmentation is prohibitively time-consuming in clinical practice. That is why the two-dimensional (2D) Macdonald criteria (7) are used; they require the longest diameter on axial slices inside the enhancement, and, subsequently, the largest perpendicular diameter has to be determined. The measurement is performed for a maximum of five target lesions and then summed up to form the sum of product of perpendicular diameters (SPD). The SPD is compared to the smallest previous tumor measurement (nadir) or the baseline. This 2D Macdonald measurement is a surrogate of the actual tumor size. How representative this surrogate is for the tumor volume is highly shape-dependent and affected by different head placements during image acquisition between follow-up scans (8–13). The current method of interval imaging lacks evidence, and further research is needed regarding the imaging biomarkers derived from this imaging practice (14). Furthermore, the current RANO guidelines have known weaknesses, e.g., with respect to pseudo-progression, which can occur until 6 months after completion of radiotherapy (15).

Advances in the field of Machine Learning (ML, e.g., Deep Learning, Artificial Intelligence) have led to a quality of automated tumor segmentation that is on par with that of expert human raters (16–19). In other words, these automated methods promise a fast and consistent assessment of the tumor volume compared to the currently used two-dimensional measurements.

A large body of work has been published comparing automated methods to manual segmentations on pre-operative MRI [e.g., (19–21)]. This study focused on publications and experiments regarding the longitudinal aspect. Most prior work on automated measurements was performed on a population level. It showed high agreement between the automated 2D SPD and volumetric measurements with expert segmentation and measurements on individual time points. In a large multi-center study, tumor volumes from HD-GLIO-AUTO segmentations showed a high agreement regarding the overlap and a promising estimate of derived time-to-progression compared to expert readings (22). Similarly, the inter-rater variability of Macdonald measurements and volume differences of a computerized method and expert raters showed a high agreement between automated and manual segmentations (23). The latter study included an automatic 2D measurement, emulating the current practice based on automated segmentation. Another similar study for pediatric brain tumors showed high agreement between automated segmentation volumes and 2D measurements and human raters on a population level (24). BraTumIA was tested by comparing its outputs to multiple expert readers on data from 14 GBM patients by evaluating the longitudinal trend disagreement (16). Another study using BraTumIA compared automated 2D measurements with volumetry on pre-operative and immediate post-operative MRI; no significant differences were found between manual and automated SPD measurements on pre-operative scans. However, for immediate post-operative use, blood depositions and reactive enhancement of structures neighboring the resection cavity degraded the quality of the SPD measurements (17).

The high agreement between the human raters and automated methods and between 2D measurements and volumetry found in these studies is encouraging regarding the applicability of automated response assessment. To transition from two-dimensional measurement to automated volumetry, accurate, robust, and longitudinally consistent, automated methods are needed alongside proven volumetric response assessment thresholds. The current RANO guidelines already contain estimated volumetric criteria, i.e., a ≥40% increase for progression (2). Since they assume isotropic growth of the lesions, they likely need further refinement and clinical validation. Furthermore, the current practice needs to be thoroughly re-evaluated regarding the lesion measurability threshold and the necessity of limiting the number of target lesions. The current guidelines are sensitive to the overall tumor burden and the lesion count of the longitudinal time points, which previous studies did not address.

We built on previous research and moved toward a view on a patient level with additional evaluations. We assessed two automated segmentation tools on the retrospective longitudinal single-center GBM dataset LUMIERE (25), which was rated according to the RANO criteria by a neuroradiologist. Based on the segmentation, we automated the calculation of the 2D SPD. To offer an alternative measurement approach to the 2D SPD measurements on axial slices, we also tested an approach where the perpendicular diameters were not restricted to the axial plane (named “2.5D” in this study).

We contribute toward automated response assessment in the following areas:

1. Longitudinal consistency is critical for reliable response assessment. Compared to expert measurements, we evaluated the patient-level trend agreement between 2D and volume measurements from automated segmentation tools. Furthermore, we assessed how the performance changes if false-positive (FP) lesions are manually excluded after automatic segmentation.
2. Tool-specific thresholds may be necessary to account for different sensitivity levels when moving from Macdonald measurements to volumetry. According to Reardon et al. (26), an appropriate threshold for progression biomarkers should be set by maximizing the correlation between the time-to-progression (TTP) and the outcome, such as the overall survival (OS). We put the current 2D progression threshold and recommended volumetric thresholds to the test for automated segmentation tools. We evaluated the TTP derived from the automated measurements, compared it to the TTP extracted from the expert rating, and evaluated the TTP–OS correlations.
3. Considering lesions below the measurability threshold is a key criterion of RANO. We assessed the ability of automated tools to find non-measurable lesions reliably.
4. The underlying segmentation technique may confound the automated 2D quantification. We checked the influence of the segmentation on the automated 2D quantification with an experiment using multi-rater segmentations.

This paper aims to answer the following questions: (a) Which aspects of response assessment already work with current automated segmentation tools? (b) Are the current progression thresholds appropriate for the tested segmentation tools? (c) Is it indicated to limit automated segmentation to five target lesions and disregard lesions below the measurability threshold? Can the automated methods reliably detect non-measurable lesions? (d) Is it clinically feasible to use these systems in a supervised mode with relatively fast correction times? The homogeneous evaluation across time and studies is an advantage of the RANO criteria. This study was also motivated by the potential for further homogenization of the response assessment using automated tools.

## 2. Materials and methods

We investigated two automated brain tumor segmentation tools: BraTumIA, a software first published in 2014 with a graphical user interface (GUI) (16–18), and HD-GLIO-AUTO, a more recently released Deep Learning–based tool, evaluated in a large multi-center study (22,27,28). A newer Deep Learning–based version of BraTumIA (DeepBraTumIA) was only trained on pre-operative MRI and would not reflect the main focus on post-operative longitudinal data in this study. Both tools use the same MRI sequences as input (T1-weighted pre- and post-contrast, T2-weighted, and T2 FLAIR), include skull-stripping and co-registration. BraTumIA segments contrast-enhanced, non-enhancing tumor, necrosis, and edema. It relies on engineered features and a Random Forest (29) model to perform a voxel-wise classification, and the output is post-processed with a dense conditional random field (30). HD-GLIO-AUTO uses a modern deep neural network approach with a U-Net architecture (31). It outputs labels for contrast-enhancing tumors and T2/FLAIR abnormalities. Both were trained on pre- and post-operative MRI and clinically validated to match the inter-rater variability between trained neuroradiologists. The decision to consider HD-GLIO-AUTO was based on its highly successful underlying architecture (U-Net) and large training dataset and the reported good generalization to other centers. Among different approaches using the same neural network structure [e.g., the winning methods of the Brain Tumor Segmentation Challenge (BraTS) in recent years and the study by Chang et al. (23)], this tool had the largest multi-center training dataset. BraTumIA uses slightly older technology but is still used for studies [e.g., (32–34)]. Its training included data from the same center we use for this work, indicating how important it is to have training data from a specific center. The additional segmentation label of BraTumIA offered no advantage in the context of this study but may be relevant for future imaging-based biomarker research. HD-GLIO-AUTO provides a faster output of the segmentation masks and a more robust deep learning–based skull-stripping algorithm. The selection of these two tools should offer insight into the relevance of center-specific training data vs. vast multi-center data and a comparison between two underlying machine learning techniques.

### 2.1. Data

The ethics committee approved the study and waived written informed consent. We retrospectively reviewed the records of 91 patients with newly diagnosed GBM who underwent pre-operative MRI between August 2008 and December 2013 and were treated with resection, followed by temozolomide-based chemoradiation, according to the Stupp protocol (35) at the Inselspital (Bern University Hospital). Patient inclusion criteria were as follows: (1) pathologically confirmed primary GBM and (2) MRI follow-up with postcontrast T1-weighted (T1c), T1-weighted (T1), T2-weighted (T2), and T2-weighted FLAIR images. Follow-ups with missing MRI sequences or heavy movement artifacts and patients without follow-up data were excluded, resulting in a study population of 80 patients with a total of 502 time points. All follow-up scans were rated adhering to the RANO criteria by an experienced neuroradiologist. A total of 129 time points contained target lesions and were measured by the expert. These 129 follow-ups were subsequently used to determine the trend agreement between measurement methods. We used all available follow-up scans for experiments that did not require expert measurements (details in Section 2.3). An anonymized version of the dataset used in this study was published as the LUMIERE dataset (25).

For automated processing, the T1, T2, and FLAIR sequences were resampled to 1 mm iso-voxels matching the resolution of the T1c images. Skull-stripping was performed within both assessed tools separately. The range of relevant MRI parameters is reported in the study by Suter et al. (25). The subsets of this data and the measurement method used in this study are described below.

### 2.2. Lesion size measurements

The size of the contrast-enhancing lesions was measured with the following four methods: (1) expert 2D measurements on axial slices according to the RANO guidelines, summed up to form the SPD, (2) automated 2D measurement, (3) automated volumetry, and (4) automated SPD measurement without the constraint for the diameters to lie on the axial plane, as an intermediate step between 2D and volumetry (2.5D). The following sections detail these measurements performed on the MRI data (by the expert) and the two automated segmentation methods.

#### 2.2.1. Expert measurements

The full dataset was rated by a neuroradiologist (14 years of experience) according to the RANO guidelines. Measurable lesions were quantified for all time points by calculating the 2D product of perpendicular diameters for the contrast enhancement on axial slices. The sum of all measured lesions per time point (up to five target lesions) was used to form the SPD. This evaluation resulted in a total of 129 time points with measurable lesions and complete imaging data. Each follow-up was classified as progressive disease, stable disease, partial response, or complete response. The time from the first resection to the first time point rated as progressive disease was used as the expert TTP. The expert did not use any prior information regarding the automated segmentation and performed the assessment based on the original un-processed MRI data. The following sections on automated measurements are based on the outputs of the two segmentation tools.

#### 2.2.2. Automated 2D measurement

The contrast-enhancing tumor segmentation was first converted into a contour on axial slices. These contours were resampled to achieve sub-voxel resolution, and the longest diameter inside the segmentation was determined through an exhaustive search across all slices. The longest perpendicular diameter was calculated with a tolerance of $90\pm 2^{\circ }$, being more restrictive than Chang et al. (23). This measurement was repeated for all separate lesions, slices with the segmentation, and found diameters were saved for inspection, and the SPD was calculated. A lesion was considered separated if its segmentation voxels did not touch the faces, edges, or other lesions’ corners.

#### 2.2.3. Automated volumetry

We quantified the contrast enhancement volume by counting the voxels of the segmentation label, considering the volume of an individual voxel.

#### 2.2.4. Automated 2.5D measurement

This measurement was a mix between the current two-dimensional method and full three-dimensional evaluation. We first found the longest diameter that lays completely inside the contrast-enhanced segmentation through an exhaustive search, not restricting this diameter to the axial plane. Afterward, a plane perpendicular to this longest diameter was moved through the segmentation. The longest diameter between any two points on the intersection curve between this perpendicular plane and the segmentation boundary fully inside the enhancement was measured. As with the 2D measurement, this was repeated for all lesions, and the 2.5D SPD was calculated. We performed these measurements on the whole dataset for both tested segmentation tools.

Technical details about the implementation are available in the Supplementary material. The code used can be found on https://github.com/ysuter/gbm-longitudinaleval.git.

### 2.3. Experiments

This study focused on the feasibility of segmentation tools for automated response assessment. This sets the primary interest on accurately tracking the contrast-enhancing tumor size and lesion count across time for individual patients. Concerning methodological tradeoffs related to vendor- and sequence-specific aspects, a segmentation method with a consistent bias could be favored over other methods that may produce more accurate labels on single time points but over- or under-segment less consistently. Hence, our evaluation was centered on evaluations showing the ability to model relative differences consistently and how the automated measurements performed within the current RANO recommendations.

As a first evaluation method, we followed a similar approach as in Meier et al.(16), calculating the trend agreement for the tested measurement methods. In short, for all patients, the measured tumor size trend was evaluated and rated as increasing, decreasing, or constant between two adjacent time points. We defined the trend agreement as the percentage of these trends that have a consensus for the tested methods across the whole dataset. We furthermore calculated Spearman’s rank correlation coefficient to evaluate the ability of a method to accurately model the ordering of values without assuming a linear relationship. In our case, this assessment focused on the tumor size ranking consistency across time points. This trend agreement and correlation were evaluated on the 129 time points where the expert rater quantified a measurable tumor burden.

**Figure 1:**
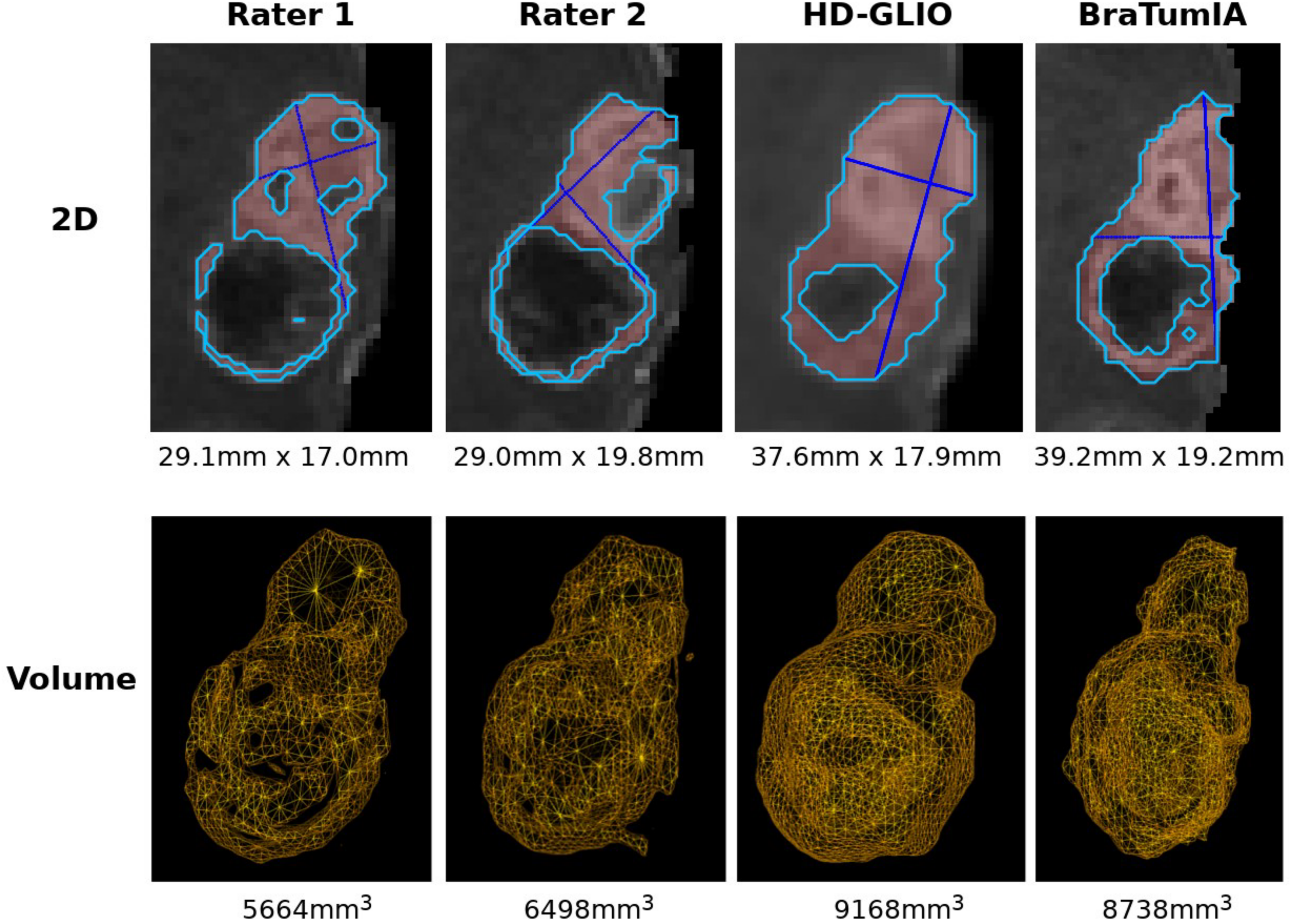
Examples of 2D and volumetric contrast-enhanced quantification. The basis for all two-dimensional quantification is a segmentation (by a human expert or automated segmentation tool) calculated automatically. 2D: the differences in the two perpendicular diameter locations show the method’s sensitivity to slight changes in tumor shape. Note that different slices are shown since the slice containing the largest diameter must be selected for the Macdonald measurement. Large differences often arise due to differences along the resection cavity. The longest line is often found touching the cavity walls and, therefore, sensitive to segmentation differences and segmenting scattered individual lesions instead of a connected boundary. Comparing the shapes in the lower row shows a smoother surface for the HD-GLIO segmentation, leading to a higher agreement between the volumetric and two-dimensional measurements (see also Figure 2).

The same analysis was repeated after manually curating 30 patients by removing false-positive lesions from BraTumIA segmentations. This patient subset was selected by considering only the patients with the highest number of complete follow-up scans. This allowed us to evaluate the trend agreement across the longest possible timespan. The measurements by the expert differ from the automated techniques in the critical point that they were not performed on a prior segmentation but by visual assessment. We, therefore, wanted to disentangle this confounding factor from the analysis by using expert segmentations as a starting point and run the automated segmentation and measurement methods for an additional comparison. We relied on data from Meier et al.(16) to perform this experiment. This multi-rater dataset consists of 14 GBM patients with 64 time points, each manually segmented by the same two experts. Furthermore, skull-stripping was manually corrected, removing another confounding effect. Due to the prior skull-stripping, we used HD-GLIO instead of HD-GLIO-AUTO, avoiding an additional brain extraction. The remaining processing steps omitted by using HD-GLIO were performed separately, such as enforcing a consistent image orientation. For the remainder of the document, we will use HD-GLIO-AUTO and HD-GLIO interchangeably for brevity. Example segmentations with the 2D and volume measurements for the automated methods and two expert raters are displayed in Figure 1.

We additionally report the multi-rater data’s intraclass correlation coefficient (ICC) (36), with a two-way, single-measurement model assessing consistency. We aimed at consistency and not absolute agreement since only relative changes are evaluated in response assessment. A constant offset of either an automated tool or an expert rater does not have an adverse effect on the response assessment. We assessed the TTP–OS correlation for the current RANO-defined progression thresholds for all measurement methods and segmentation tools to evaluate the current progression thresholds. Furthermore, we report the difference between the automatically obtained TTP and the expert TTP.

## 3. Results

This section presents the results and a brief discussion for each aspect tested with an experiment.

### 3.1. Longitudinal trend agreement with 2D expert SPD

**Figure 2:**
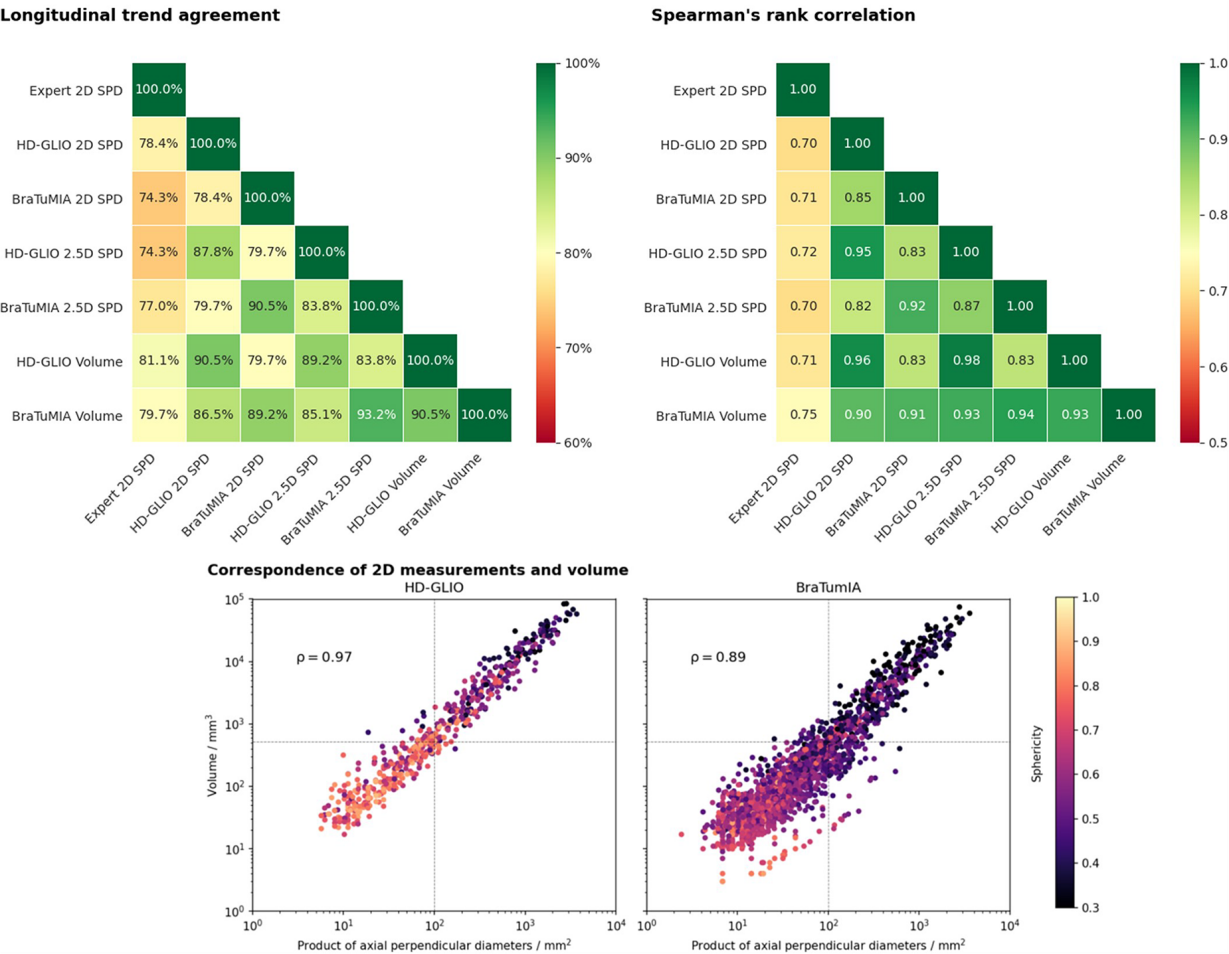
Top row: Longitudinal trend agreement (left) and Spearman’s rank correlation coefficient (right) for the studied automated measurement methods and measurements by an expert. The trend agreement is calculated by comparing the slope between consecutive follow-ups. The highest trend agreement between the expert SPD and an automated measurement was achieved for the volume derived from the HD-GLIO segmentation (81.1%). Overall, the highest trend agreement observed was between the 2.5D measurement and volume of the BraTumIA segmentation. The agreement between the expert rater and both automated tools was generally lower than between the automated methods. Spearman’s rank correlation shows a very similar pattern. The volume from the BraTumIA segmentation had the highest correlation to the expert measurement. Bottom: Correspondence between 2D measurements and the volume of individual lesions. Dashed line: 2D size and volume of a sphere with 10 mm diameter (2D RANO measurability threshold). Lesion segmentations from HD-GLIO were more spherical, explaining the better 2D–volume correspondence and high agreement between the 2D and volumetric trend. In addition, HD-GLIO segments show fewer unconnected lesions when compared to BraTumIA, visible by the fewer data points in the plot.

Figure 2 shows the results for the trend agreement analysis and Spearman’s rank correlation. The HD-GLIO segmentations’ volume reached the highest trend agreement of 81.1% with the expert SPD measurements, followed by the BraTumIA volume. The 2.5D measurement was a better surrogate for the volume than the 2D method for BraTumIA, but not HD-GLIO. The agreement considering a specific tool was high for all tested measurement methods. If only the agreement within a given software was considered, all measurement methods agreed on 89.2% of the trends. The results for Spearman’s rank correlation show the same pattern. The correlation between the expert SPD and an automated method was highest for BraTumIA’s volume, followed by the 2.5D SPD from HD-GLIO. Visual assessment of the 2.5D longest diameter’s direction showed inconsistent orientations between follow-up time points, leading to unstable results. This variability indicates that using axial slices for the 2D SPD has a regularizing effect by reducing the degree of freedom, even though the head placement may differ across scans.

HD-GLIO showed a higher agreement between the 2D SPD and lesion volume. This 2D–volume correspondence is closely linked to the lesion shape. The bottom of Figure 2 shows the sphericity for individual lesions. We observed more spherical lesions for HD-GLIO than for BraTumIA. For BraTumIA, lesions were often divided into smaller lesions, especially during recurrence along the resection cavity wall. Spearman’s rank correlation between 2D and volumetric measurements was 0.97 for HD-GLIO and 0.89 for BraTumIA. In addition, HD-GLIO segmented fewer separate lesions (note the number of data points in the plot).

The results for the 2.5D measurements are listed in the Supplementary material.

### 3.2. Time-to-progression based on automated segmentations

**Figure 3:**
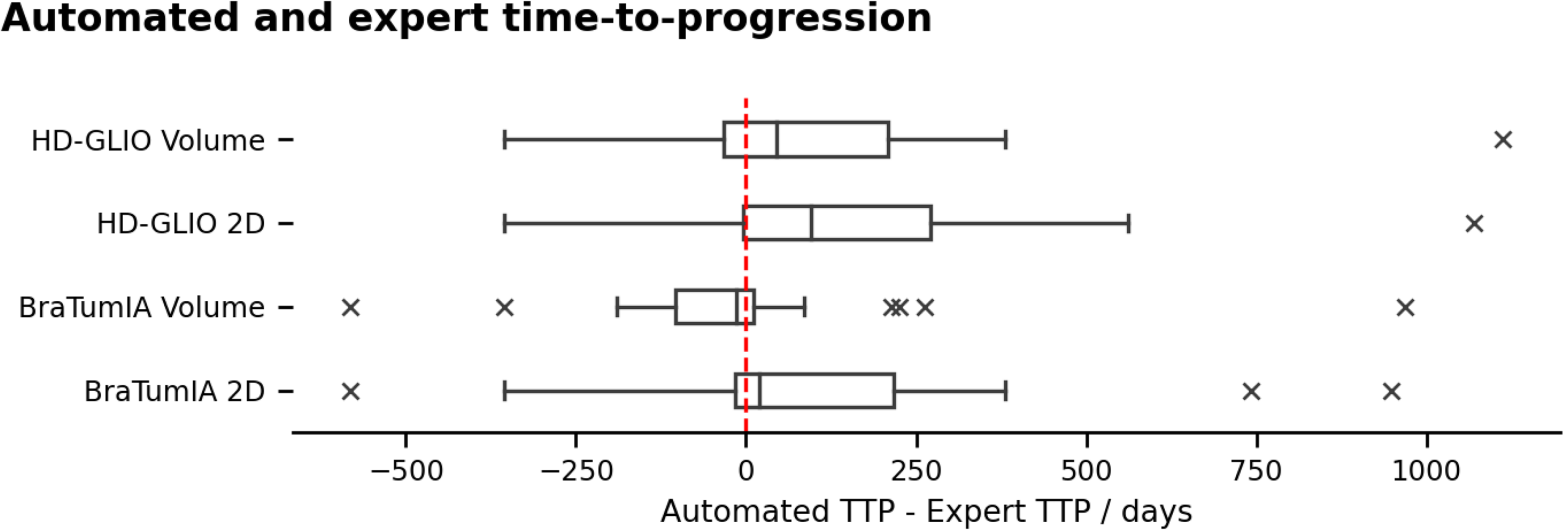
TTP calculated based on the automated segmentation and the RANO progression thresholds, compared to the expert rater. Negative range: under-estimation of the TTP by the automated methods. Outliers in the positive range were caused by under-segmentation of the contrast enhancement, preventing some patients from reaching the progression threshold. The TTP was set to the overall survival time for these cases.

Figure 3 and Table 1 compare the TTP derived from the expert ratings and apply the current 2D SPD and volume progression thresholds from the RANO guidelines (≥25% 2D, ≥40% volume increase). We report the results as the difference between the automated TTP and expert TTP. BraTumIA segmentations’ volume reached the closest mean match between the expert TTP and automated measurements (−9.75 days), followed by the 2D SPD from the same tool. The same software and measurement method reached the lowest interquartile range (144.5 days). Extreme outliers where the automated TTP was far longer than the expert TTP were caused by under-segmentations not reaching the progression threshold at any time point, and the TTP was set to equal the OS time. We note that, on average, the volume progression threshold was detected before the 2D SPD for both segmentation methods. We used the follow-up time points identified as the nadir/baseline for these results since severe under-segmentation would have led to wrongly reassigning the reference time point when relying on the automated segmentation. Spearman’s rank correlation between TTP and OS was highest at 0.55 ($p=8\times 10^{-7}$) for the HD-GLIO 2D SPD and lowest for the BraTumIA volume at 0.31 (p=0.008). The expert TTP–OS correlation was 0.4 ($p=6\times 10^{-4}$).

**Table 1:** Comparison of the TTP based on automated segmentations and the expert-rated TTP.

	Automated TTP—Expert TTP			TTP+-OS correlation	
Method	Mean ± SD (days)	Median (days)	IQR (days)	Correlation	p-value
HD-GLIO volume	93.0±273.8	45.0	241.5	0.48	$3\times 10^{-5}$
HD-GLIO 2D SPD	142.5±290.3	95.5	274.0	0.55	8$\times 10^{-7}$
BraTumIA volume	−9.8±267.4	−14.5	114.5	0.31	0.008
BraTumIA 2D SPD	92.5±304.5	20.0	233.3	0.44	$1.7\times 10^{-4}$
Expert 2D SPD	—	—	—	0.40	$6.3\times 10^{-4}$

IQR, interquartile range.

This table shows the difference between automated and expert TTP. Both the volumetric and 2D progression thresholds were applied. Figure 3 shows the same data as a boxplot. The BraTumIA measurements showed the highest agreement with the expert TTP, but all tested methods showed considerable variability. 2D SPD from the HD-GLIO segmentation had the highest correlations between TTP and OS.

### 3.3. Data-driven progression thresholds

**Figure 4:**
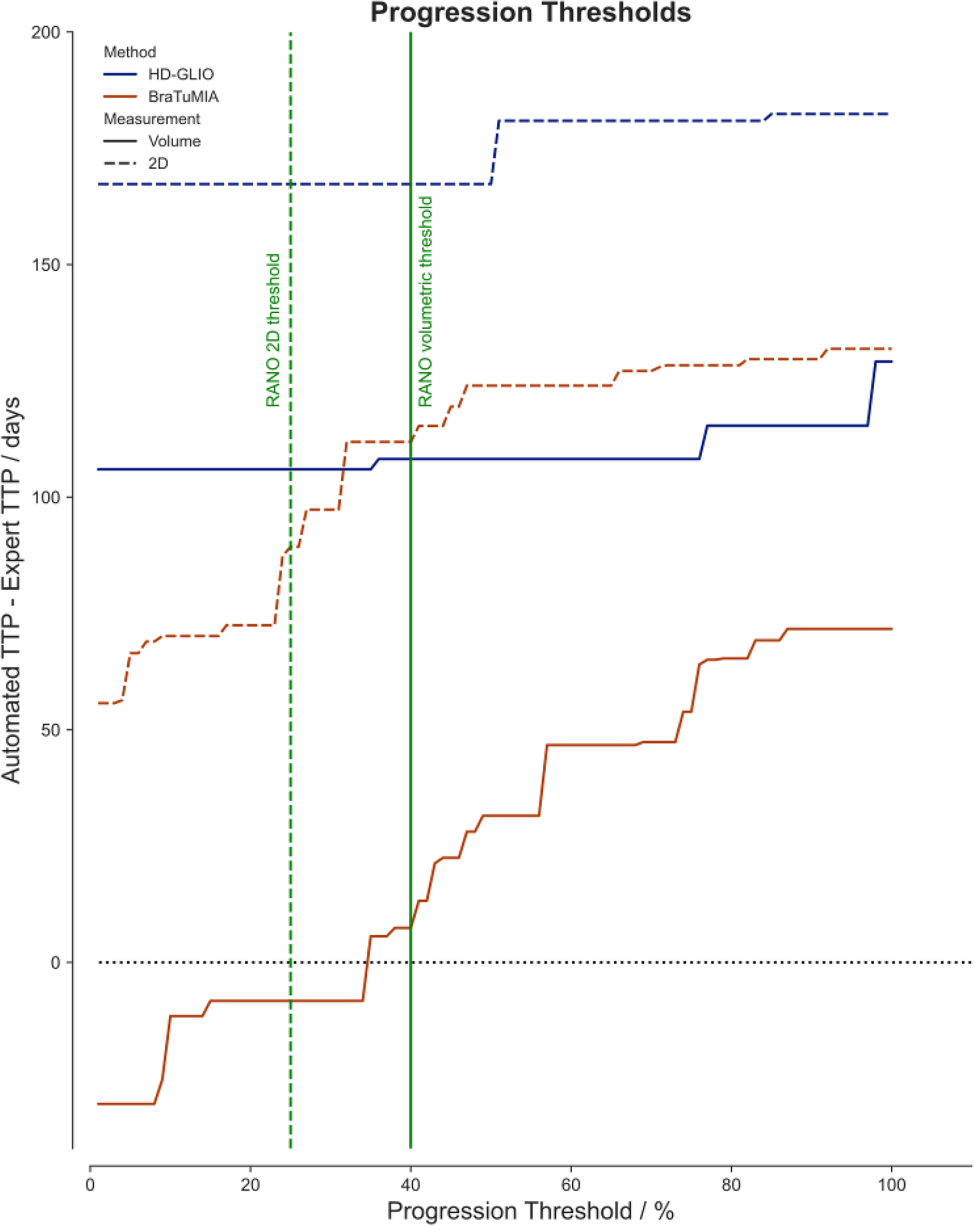
The mean difference of the automated TTP to the expert rater for different progression thresholds. The solid lines visualize the data for the volumetric measurements based on the HD-GLIO (blue) and BraTumIA (red) segmentations. The dashed lines show the automated 2D measurements. The current progression thresholds are shown in green for 2D measurements (dashed line, at 25%) and volumes (solid line, at 40%).

Based on the findings of the differences in the TTP between the expert and the automated tools, we evaluated if the progression thresholds could be adapted to the sensitivity of a given tool. Figure 4 shows the mean TTP difference to the expert for both tools and measurement methods for different thresholds. For HD-GLIO, progression would have been detected, on average, too late even for an arbitrarily low progression threshold. The volume measurement by BraTumIA was the only method where progression could have been caught on time, on average, with a slightly lower threshold. For both HD-GLIO and BraTumIA, the 2D measurements would have resulted in a delayed progression classification.

### 3.4. Longitudinal trend agreement with manual FP removal

**Figure 5:**
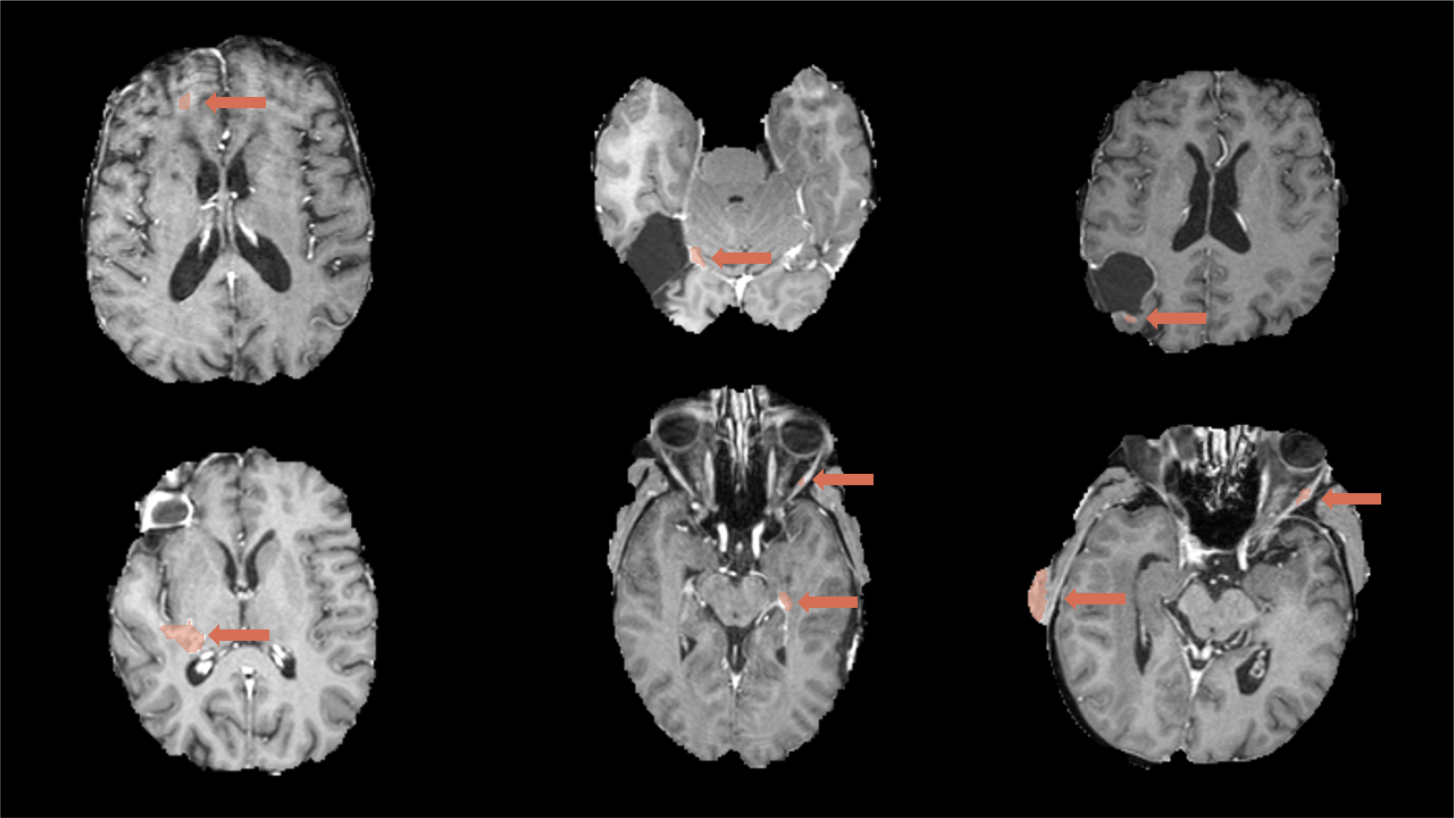
Examples of false-positive segmentations by BraTumIA that were removed during the manual correction, shown together with the post-contrast T1-weighted image. Top left: false positives potentially caused by image artifacts; top middle and right: false positives along the resection cavity; bottom left and middle: distant false positives including vessels; bottom middle and right: false positives caused by incomplete skull-stripping.

**Figure 6:**
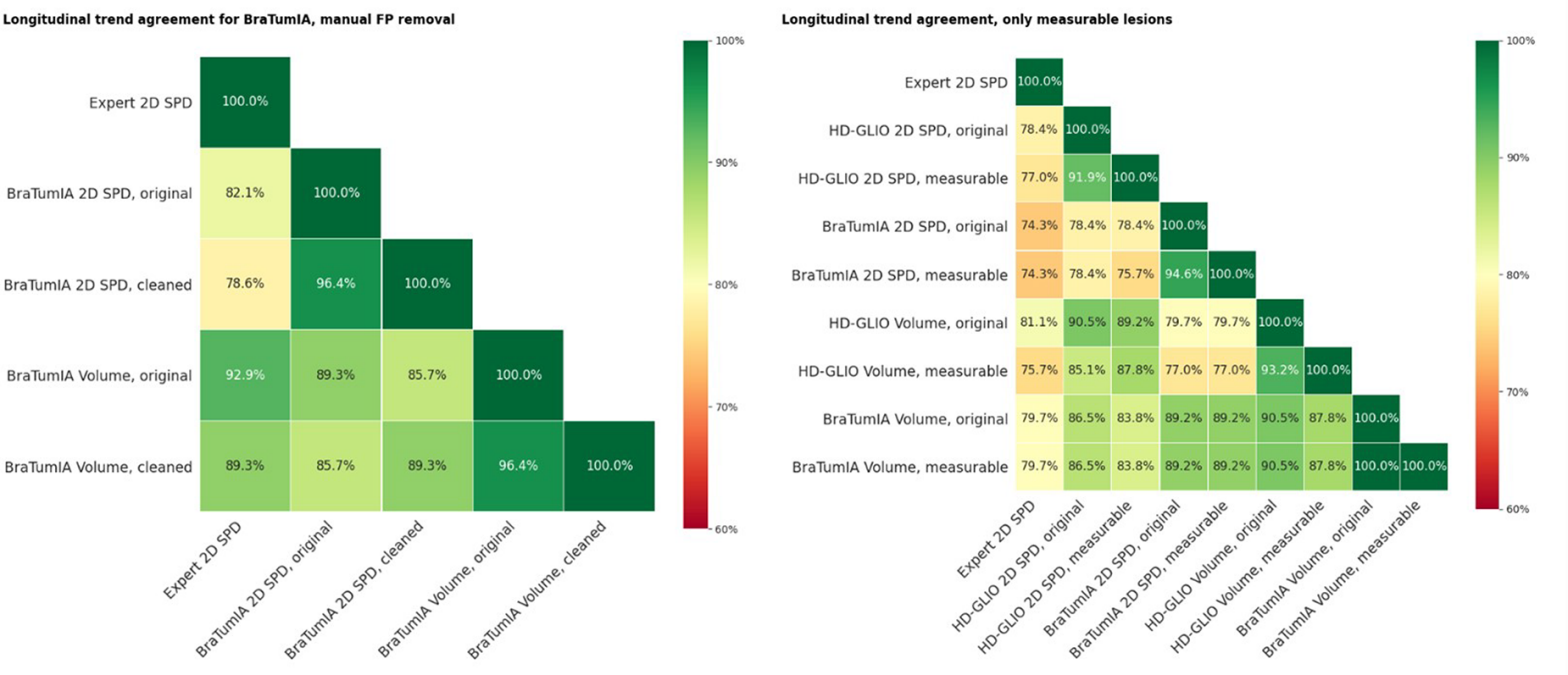
(Left) Trend agreement for a subset of 30 patients for measurements based on BraTumIA segmentations, where FP were removed manually. Original: unedited segmentation; cleaned: false-positive lesions removed. The trend agreement for automated methods compared to the expert measurements slightly decreased, indicating that this correction step is not sufficient. (Right) Trend agreement between the expert and automated methods, where non-measurable lesions were automatically removed and limited to a maximum of five.

We observed many false-positive lesions, especially for BraTumIA (in 64.5% of inspected time points), primarily for images with insufficient brain extraction. Figure 5 shows examples of false-positive lesions encountered during inspection of the results. For the un-processed BraTumIA measurement, the agreement with the expert 2D SPD was 82.1% for the 2D measurement and 92.0% for volumetry. For both quantification methods, agreement with the expert decreased after FP removal to 78.6% for the 2D SPD and 89.3% for the volume. So even for a more sensitive method, manual FP removal was insufficient to boost the performance regarding the trend agreement, calling for manual corrections of missed lesions. Figure 6 (left) displays these results.

### 3.5. Longitudinal trend agreement emulating RANO requirements—effect of measurability threshold and number of quantified lesions

We always considered all segmented lesions for the results presented up to this point. The current RANO guidelines limit to a maximum of five measurable lesions and a 2D measurability threshold of 10 mm of both bi-dimensional diameters for 1mm iso-voxels. Especially for lesions recurring at resection cavity walls, the automated tools often split lesions into smaller parts; a human rater would consider a single connected lesion. This impacted the automatic measurements in two ways: smaller lesions may fall below the measurability threshold. If not, multiple lesions are considered to build the SPD, leading to a different value. We repeated the same trend agreement analysis in Figure 6 (right), excluding lesions below the 2D measurability threshold and limiting the maximum measurable lesion count to the five largest lesions. When comparing the automated segmentations to the expert 2D SPD, excluding non-measurable lesions had a detrimental effect on the HD-GLIO output measurements but did not change the trend agreement for BraTumIA. The trend agreement with the expert was lowered by 1.4% for the 2D SPD and by 5.4% for the volume from HD-GLIO after removing non-measurable lesions. This indicates that, for automated segmentation, filtering out small lesions can be detrimental compared to the currently established 2D expert measurements.

### 3.6. Longitudinal trend agreement for multi-rater data

**Figure 7:**
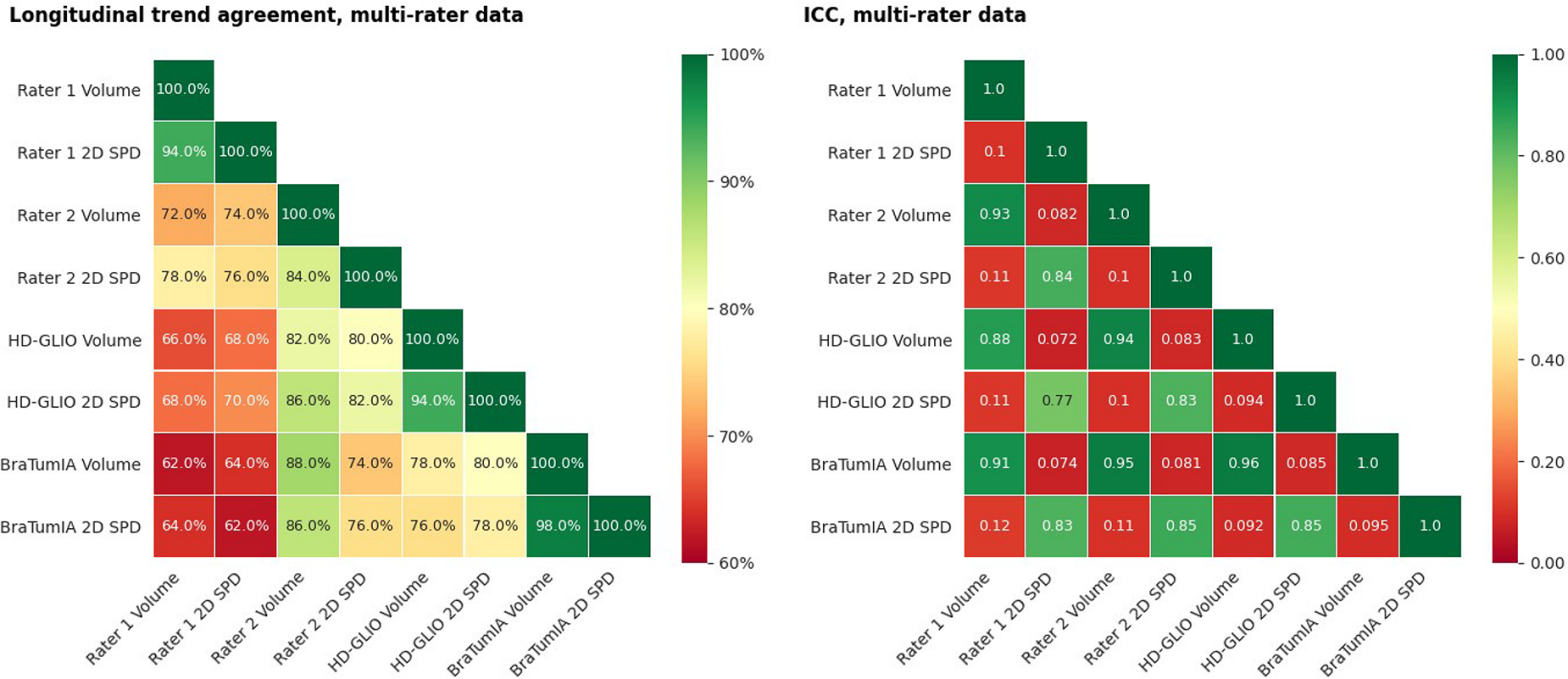
(Left) Trend agreement for automated methods and measurements of segmentations by two human raters. This experiment is based on data from an earlier study, including 14 patients with a total of 64 time points. The trend agreement between automated methods was higher than between the two experts. The agreement between the first rater and both automated methods was lower than the inter-rater trend agreement. (Right) ICC for the same data. The ICC was calculated based on consistency, two-way mixed-effects and a single-measurement model.

Two apparent factors have confounded the previous experiments: (a) The expert 2D measurement was performed without prior segmentation of the contrast enhancement, while the basis for the automated measurements was a segmentation; and (b) only time points with measurable lesions were considered. Therefore, the following results are a more targeted analysis of the impact of measurement techniques. The starting point was a segmentation, either by a human expert or the output of an automation tool. The results show that the automated segmentations had a similar trend agreement within themselves as the inter-rater agreement for both volume and 2D SPD. The volume trend agreement between the human raters lay at 72% and 78% for the automated tools. The 2D SPD derived from the segmentations agreed with 76% for the human raters and 78% for the automated methods. Rater 1 had a low agreement for both volume and 2D SPD to all automated methods. Visual inspection showed that the high 2D disagreement stemmed from scattering lesions into unconnected parts the other rater and automated tools had detected as one connected lesion. The results are shown in Figure 7.

### 3.7. Non-measurable lesions

To add another element toward automated response assessment, we compared the number of time points with non-measurable lesions detected by the expert rater to BraTumIA and HD-GLIO. The expert recorded non-measurable lesions in 45 out of 385 post-operative follow-up scans. Out of these 45 time points, HD-GLIO also found non-measurable lesions in 20 time points and BraTumIA in 30 time points. For the 340 time points without non-measurable lesions according to the expert rating, HD-GLIO still segmented non-measurable lesions for 94 time points and BraTumIA for 200 time points. To check for a constant offset of the automated segmentation tools, we compared the fraction of non-measurable lesion size of the full tumor burden for the automated 2D and volume measurements for time points with and without expert-rated non-measurable lesions but did not find a significant difference (Wilcoxon signed-rank test).

## 4. Discussion

The tested tools reached high trend agreements with an expert rater following the RANO guidelines. Even the highest reported trend agreement with the human rater of 81.1% (HD-GLIO volume) is still insufficient to be used without expert input for clinical decision support. Failure to detect a large lesion would lead to a wrongly updated nadir time point.

Since the current RANO guidelines list the appearance of new non-measurable lesions as a progression criterion, the lesion count needs to be accurate. Still, it was highly inconsistent across time, especially for post-operative time points. The main issue is the automated methods segmenting recurrence along resection cavity walls as many separate lesions when a human rater would identify a single lesion. This was especially problematic when the 2D SPD was calculated from automated segmentations since many smaller lesions were measured instead of a single lesion. This heavily distorted the 2D measurements but not the automated volumetry. Both automated segmentation tools failed to detect all expert-rated non-measurable lesions. The issue of fractioning leads to many time points where the expert recorded only measurable lesions, but the automated tools segmented small non-measurable lesions.

The simple removal of false-positive lesions even had an adverse effect, making more complex corrections necessary. BraTumIA produced more false-positive lesions than HD-GLIO but had a clear edge when individual lesions had to be tracked longitudinally. The tested segmentation tools’ main challenge remained false positives due to incorrect skull-stripping on post-operative scans, an observation made in other studies (19).

The training set of BraTumIA contained GBM data from our center. So despite relying on less recent technology, it can compete closely with HD-GLIO, trained on a large multi-center dataset. We hypothesize that center-specific training can remarkably lift the performance of such tools, and a balance has to be found between center-specific performance and generalizability. With these results, we would like to encourage developers of automated segmentation tools to include the ability for users to continue training with their data at the target center while still benefitting from a more extensive and diverse training data set.

Picking up the listed research questions from the introduction, we conclude with the following.

### 4.1. Suitability of automated segmentation tools for response assessment

Despite the relatively high trend agreement, the automated tools under- and over-segmented few time points. Apart from a falsely detected progression or response, this may lead to a nadir re-assignment if we rely entirely on the automated output. Therefore, we recommend the current automated tools as a time-saving aid for neuroradiologists with close monitoring and manual corrections where needed.

### 4.2. Progression thresholds

The results indicate good applicability of the current thresholds for 2D SPD and volumetry for BraTumIA. For HD-GLIO, progression was detected too late on average. We recommend adjusting progression thresholds to the sensitivity and specificity of each segmentation method. This adjusted tool-specific method should be validated on large multi-center datasets and could be complemented by maximizing the TTP–OS correlation as proposed by Reardon et al. (26). A challenge will be the confounding factor that current progression ground truth RANO ratings rely on 2D SPD measurements, being a poor surrogate for the actual tumor burden for complex tumor shapes. In our first analysis, we found that it was possible to match the expert TTP with a custom progression threshold for volumetric assessment with BraTumIA.

### 4.3. Lesion measurability for automated segmentations

Our experiments showed a lower trend agreement with the expert measurements when only lesions above the 2D measurability threshold and a maximum of five target lesions were considered. While the 2D measurements have a practical lower size limitation to 10 mm in both axes, we recommend considering all detected lesions for both tested automated tools.

### 4.4. Manual corrections

Removing false-positive lesions on the BraTumIA output was insufficient, and human input should correct for under- and over-segmentation. This analysis was omitted for HD-GLIO since it produced very few false positives. Our findings show that automated segmentation can be a time-saving aid to enable clinicians to move toward response assessment based on the contrast-enhancing volume. Automatically calculating the 2D SPD from segmentations may help facilitate the transition to volumetry to understand differences, especially in the context of well-established progression thresholds and careful evaluation of irregularly shaped or scattered recurrence along resection cavity walls. Further development is needed to enable automated evaluation of treatment response beyond tumor size, particularly for robust and longitudinally consistent segmentation of non-measurable lesions. Human correction and safeguards remain key when used in clinics with the currently available methods.

We note that we did not test any automated post-processing methods outside of the tested tools to assess their current capabilities.

### 4.5. Limitations

Our study has a few limitations, requiring more validation and research. First, our findings are based on a single-center retrospective analysis with a small sample size, and further confirmation from independent cohorts and different centers with varying acquisition and follow-up protocols are needed. While the imaging adhered to the current International Standardized Brain Tumor Imaging Protocol (2), the acquisition parameters were heterogeneous, and how this compared to the distribution of the training data used for the investigated tools is unclear. Treatment differences and group differences in molecular markers may confound the correlation analysis of tumor measurements with the overall survival time. Due to the time-consuming nature of manual tumor measurements, only one expert rater performed the Macdonald/RANO measurements, except for the multi-rater data, where we could rely on manual segmentations from two experts. Chang et al. (23) report an ICC of 0.704 between two independent raters for the 2D measurements. This is similar to the ICC values we found between the automated methods and the human rater and between the automated tools. We, therefore, would not expect considerable changes in our findings if more experts were included or if a consensus read had been sought.

We did not include evaluation confidence intervals since we relied on simple aggregated scores such as the trend agreement. Studies with a larger sample size and more elaborate analysis should already consider confidence metrics in the study design.

### 4.6. Outlook

We see the longitudinal consistency regarding lesions identified, accurately counted, and tracked as the key development step needed for automated segmentation tools. A potential research direction could include information on previous follow-ups or pre-operative scans in the segmentation pipeline. An intermediate step could be to incorporate post-processing steps, e.g., based on distance metrics from the pre-operative segmentation and follow-up information. For wide clinical adoption, automated segmentation tools should ideally offer tools for easy manual correction and review of previous time points. Making co-registration of follow-up imaging a standard for the 2D measurement can alleviate inconsistencies and improve the measurement homogeneity. Tools should include basic checks if the input images are of sufficient quality and fit within the training data distribution. Our performance comparison suggests that re-training models trained on multi-center imaging on center-specific data should be considered and facilitated. Once sufficient longitudinal consistency is achieved, tool-specific progression thresholds should be evaluated on ideally publicly available multi-center benchmarks. Further research should go into providing confidence indicators to the users when these tools are adopted in clinical decision support and uncertainty on longitudinal data.

## Data Availability

Publicly available datasets were analyzed in this study. These data can be found here: https://figshare.com/collections/The_LUMIERE_Dataset_Longitudinal_Glioblastoma_MRI_with_Expert_RANO_Evaluation/5904905.
