# Supplementary material for: Evaluating automated longitudinal tumor measurements for glioblastoma response assessment
Source: Front Radiol. 2023 Sep 7;3:1211859. doi: 10.3389/fradi.2023.1211859 (PMC10513769; doi:10.3389/fradi.2023.1211859)
Supplement: Supplementary file 1 [file Datasheet1.pdf]

Supplementary Material

Automated 2D and 2.5D Tumor Size Measurements

The GitHub link to the automated bi-dimensional measurement code is referenced in the main document. The 2D measurement is implemented as follows: For every axial slice, the contrast-enhancement tumor segmentation is converted to a contour that is resampled to sub-voxel resolution. All pairwise distances of the contour points are calculated. Starting with the longest distance, we check if the line connecting the contour points fully lies within the segmentation mask (with the same sub-voxel resolution as before). As soon as such a line is found, we move to the next slice. We repeat this for every axial slice and retain the longest diameter found. As soon as the longest line fully inside the segmentation is found, we filter the remaining contour points of the given slice to only include point pairs with perpendicular connecting lines (± 2°). We again check for the longest such line inside the segmentation mask. This is repeated for all connected components of the segmentation mask.

For the 2.5D measurement, we extract all contour points. All pairwise distances are calculated (not restricted to axial slices) and checked for lines fully inside the segmentation mask. The resulting intersection contour is resampled and each pairwise distance calculated and checked for a connecting line between these points. As soon as such a line is found, we iterate along this line and intersect the contour with a perpendicular plane. After the iteration over the longest line is complete, we calculate our 2.5D measurement by the product of this longest line and the longest line between points on the intersection contour inside the segmentation.

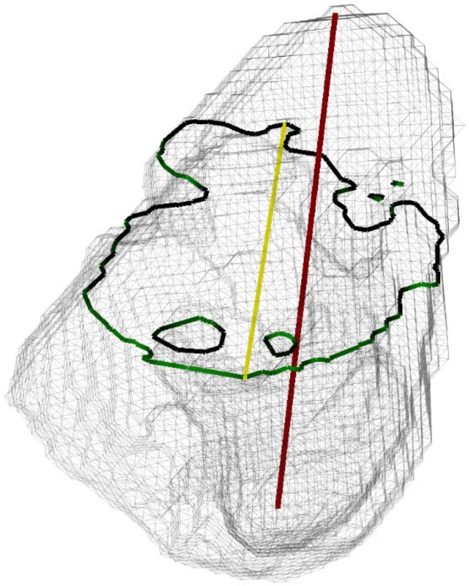

Figure S1 Illustration of the 2.5D measurement. The red line is the longest line between any point on the segmentation surface fully inside the segmentation mask. The green contour is the perpendicular intersection with the longest line (yellow) on that intersection plane perpendicular to the red line.

Automated 2D and 2.5D Tumor Size Measurements

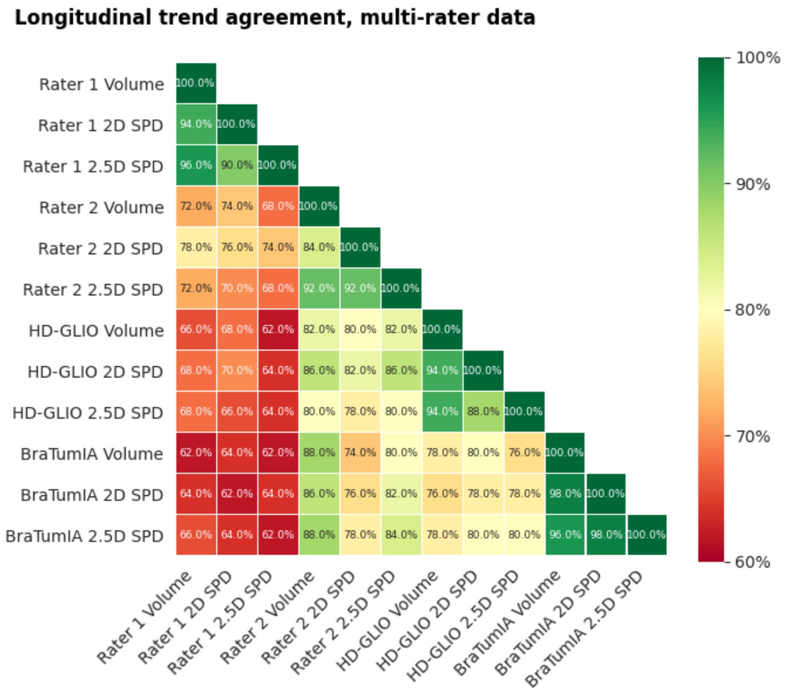

Figure S2 Longitudinal trend agreement for the multi-rater data. As in the corresponding figure in the main text but containing the 2.5D measurements.

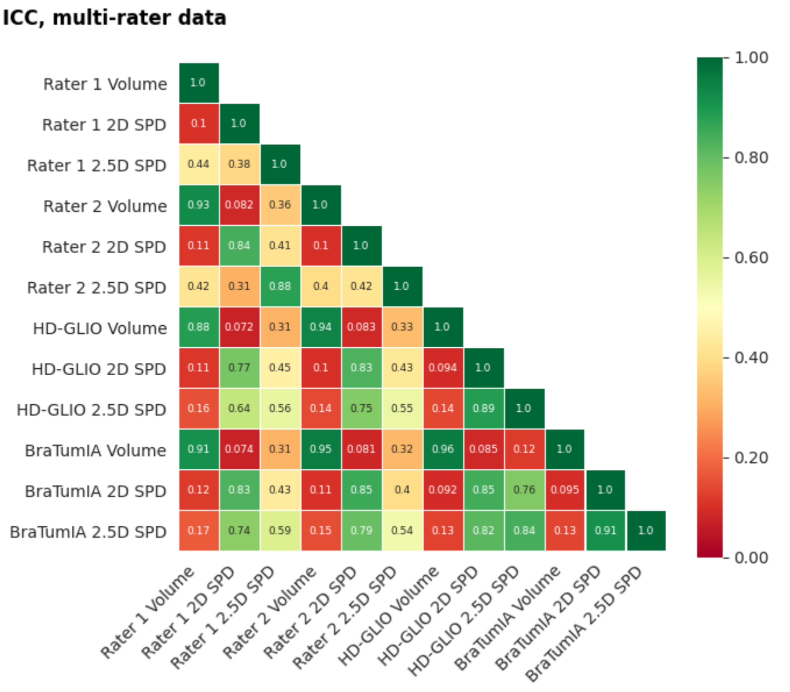

Figure S3 ICC of the multi-rater data for different measurement methods. Same as in the main text, additionally showing the 2.5D results.
